# Magnitude of the 8.2 ka event freshwater forcing based on stable isotope modelling and comparison to future Greenland melting

**DOI:** 10.1038/s41598-021-84709-5

**Published:** 2021-03-09

**Authors:** Wilton Aguiar, Katrin J. Meissner, Alvaro Montenegro, Luciana Prado, Ilana Wainer, Anders E. Carlson, Mauricio M. Mata

**Affiliations:** 1 Laboratório de Estudos dos Oceanos e Clima, Instituto de Oceanografia, Universidade Federal do Rio Grande–FURG, Rio Grande, RS 96203-900 Brazil; 2 Climate Change Research Center and ARC Centre of Excellence for Climate Extremes, University of New South Wales, Sydney, Australia; 3 Department of Geography, The Ohio State University, Columbus, OH 43210 USA; 4 Instituto Oceanográfico, Universidade de São Paulo, São Paulo, 05508-120 Brazil; 5 Oregon Glaciers Institute, Corvallis, OR USA; 6 Instituto de Geociências, Universidade de Brasília, Brasília, 70297-400 Brazil

**Keywords:** Ocean sciences, Physical oceanography, Climate sciences, Palaeoceanography, Palaeoclimate

## Abstract

The northern hemisphere experienced an abrupt cold event ~ 8200 years ago (the 8.2 ka event) that was triggered by the release of meltwater into the Labrador Sea, and resulting in a weakening of the poleward oceanic heat transport. Although this event has been considered a possible analogue for future ocean circulation changes due to the projected Greenland Ice Sheet (GIS) melting, large uncertainties in the amount and rate of freshwater released during the 8.2 ka event make such a comparison difficult. In this study, we compare sea surface temperatures and oxygen isotope ratios from 28 isotope-enabled model simulations with 35 paleoproxy records to constrain the meltwater released during the 8.2 ka event. Our results suggest that a combination of 5.3 m of meltwater in sea level rise equivalent (SLR) released over a thousand years, with a short intensification over ~ 130 years (an additional 2.2 m of equivalent SLR) due to routing of the Canadian river discharge, best reproduces the proxy anomalies. Our estimate is of the same order of magnitude as projected future GIS melting rates under the high emission scenario RCP8.5.

## Introduction

Greenland ice-sheet melting is one of the major responses to the rising atmospheric greenhouse gas concentrations and global mean temperature^1–3^. The addition of ice-sheet meltwater to the North Atlantic will potentially have a destabilizing effect on the Atlantic Meridional Overturning Circulation (AMOC), which could weaken by more than 70% within the next few centuries^4–6^. Past meltwater-driven AMOC slowdowns have repeatedly led to millennia-long cold events in the Northern Hemisphere: for example, the Oldest and Younger Dryas (~ 19 to 14.7 kiloyears and 12.9 to 11.7 kiloyears before the present, respectively)^7–9^. However, the cold event 8.2 kiloyears before present (8.2 ka event hereafter) differs from previous cold events due to its short, century-long duration^10,11^. The 8.2 ka event also took place in the current interglacial period under boundary conditions that were closer to pre-industrial conditions than earlier cold events^12^.

Several freshwater forcing hypotheses involving the Laurentide Ice Sheet (LIS) have been suggested for the 8.2 ka event. These scenarios include three freshwater sources: the drainage of Lake Agassiz^13^, the change in North American continental freshwater routing from LIS retreat^14,15^, and the on-going retreat of the LIS and its associated meltwater production^16–18^. The first two sources have relatively well-constrained discharge rates and volumes^19,20^ when compared to the direct ice-sheet meltwater source^16,17,19,20^. Even though the outburst of Lake Agassiz is commonly considered the main trigger for the 8.2 ka event^13^, recent studies have found that both the LIS retreat and change in the routing of continental discharge might have had a significant role in causing the climate event’s anomalies^15,16,21^, thus raising uncertainties on the role of each of the three meltwater sources in triggering the 8.2 ka event.

The range of estimates of the magnitude of total freshwater release during the 8.2 ka event is also large^10,11^, ranging from 1.5  to 9 m in equivalent sea-level rise (SLR)^16,22^. Some of these scenarios were previously used to simulate the cold event with numerical climate models in an attempt to estimate the climatic impacts of the freshwater discharge^23–25^, and simulation skill was evaluated by comparison with sea surface temperature (SST) reconstructions. Such a large range of meltwater volume is enough to create scenarios ranging from a small change in circulation to a total collapse of the AMOC^26^. Furthermore, due to a model-dependent stability of ocean overturning, location of deep convection sites and meridional heat transport, the simulated SST response to freshwater forcing varies significantly between distinct simulations^27,28^. This model dependency makes it difficult to test which freshwater source played the dominant role in triggering the 8.2 ka based on the comparison between the simulated SST response and reconstructed SST changes. However, these uncertainties can be reduced by using a combination of active and non-active tracers, such as oxygen isotopes as well as SSTs.

Constraining the amount of freshwater involved in the 8.2 ka event, and the role of each freshwater source in creating the climate anomalies of the event, will enhance our understanding of the sensitivity of the climate system to freshwater fluxes, which is of obvious importance for future scenarios given the observed recent acceleration of Greenland ice-sheet (GIS) mass loss^1,2,6^. In this study, we aim to constrain the magnitude and length of freshwater flux that caused the 8.2 ka event. We explore this question by using numerical simulations that calculate both oxygen isotopes, in seawater, in carbonates, and in ice cores, and SSTs prognostically and by comparing the simulations with paleoclimate records of the same variables.

## Simulations based on earlier reconstructions

The simulations presented in this section are based on different freshwater release processes that have been suggested in previous studies^15,18,22,30^. FWpe simulates a scenario where the estimated LIS melting is released exclusively into the Labrador Sea^29^; FWca represents a scenario where the Canadian continental runoff discharges into the Labrador Sea^15^; FWul simulates the melting of the remaining LIS after the collapse of the Hudson Bay’s ice saddle^22^, and FWli simulates a fast rise in sea level surrounding the 8.2 ka event due to prolonged drainage from Lake Agassiz^18^ (see “Methods”, “Freshwater forcing for the simulations based on earlier reconstructions” section). SSTs and δ^18^O anomalies from these simulations are then compared with proxy data from 27 locations, at the model’s grid cell closest to the geographical coordinates of each core (see “Methods”).

**Figure 1:**
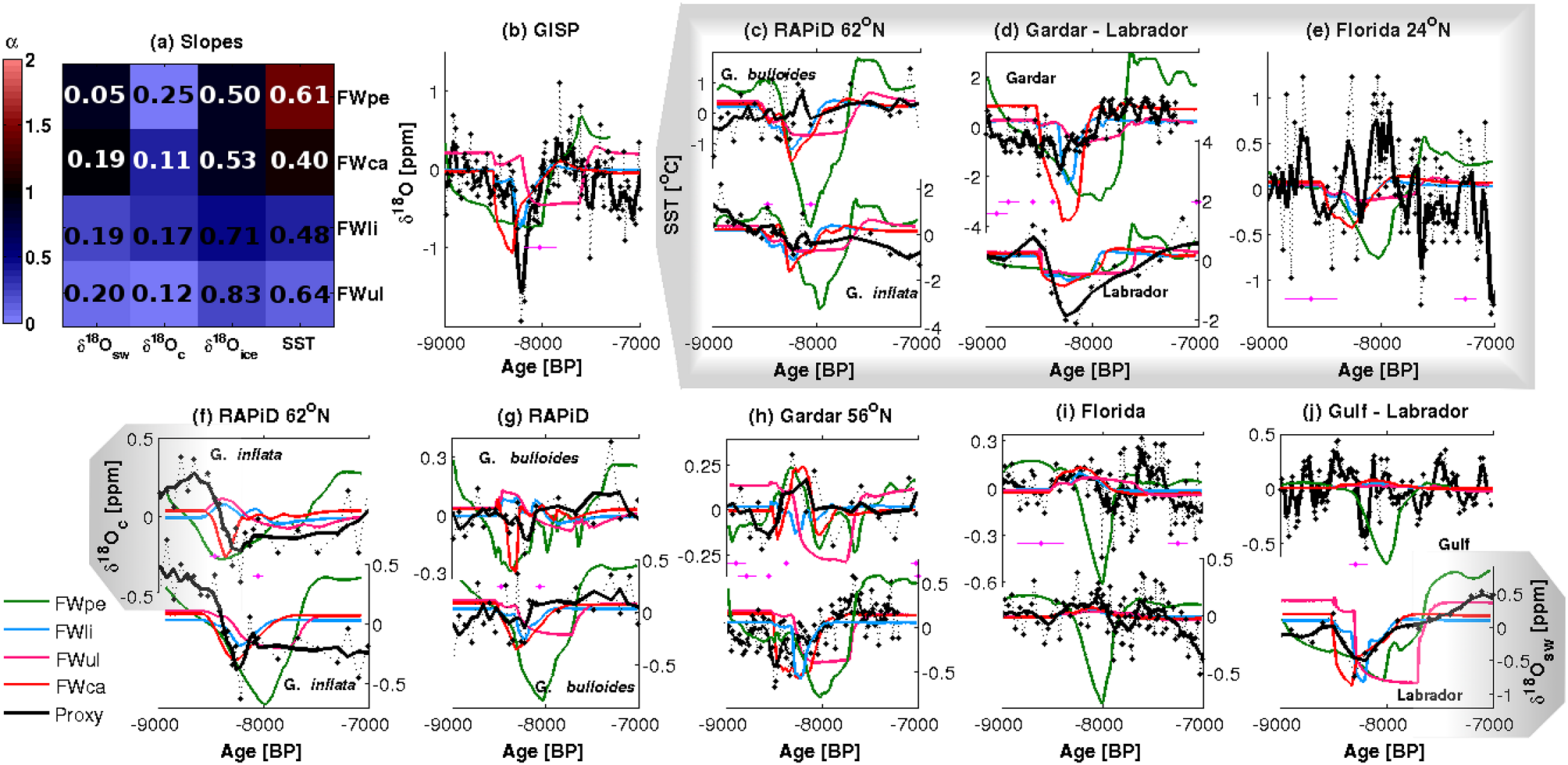
Comparison between time series of proxies and simulations for δ^18^O_sw_, δ^18^O_ice_, δ^18^O_c_ and SST for RAPiD (**c**,**g**), Gardar Drift (**d**,**h**), Florida Strait (**e**,**i**), GISP and Gulf cores (**b**,**j**), and slopes and RMSEs (**a**) in all simulations (locations in Fig. S1 and Table S1). Black dashed and full lines are core values and 2-point moving averages, respectively. Green, blue, magenta and red lines are time series for FWpe, FWli, FWul and FWca. The pink horizontal crosses are the dating ( ) and dating errors ( ) for the proxies. In (**a**) RMSE values are plotted in the center of the image while the colors of the squares indicate the values of the slopes*.* From (**f**–**j**), top series are for δ^18^O_c_, while bottom series are for δ^18^O_sw_.

Linear regression slopes and RMSEs for simulated tracers (Fig. 1a) show that FWpe and FWca yield the best estimate of δ^18^O_sw_ and δ^18^O_ice_ (${\upalpha}_{sw}^{pe}=0.89$, ${\upalpha}_{sw}^{ca}=0.99, \;\; {\upalpha}_{ice}^{pe}=0.86$ and ${\upalpha}_{ice}^{ca}=0.85$). However, FWpe overestimates SST anomalies and estimates a decrease in δ^18^O_c_ while proxy records point to an increase during this period (${\upalpha}_{sst}^{pe}=1.43$, ${\upalpha}_{c}^{pe}=-0.4$). The FWpe simulation represents the total amount of LIS melting during this period of time, however this flow did not go entirely into the Labrador Sea^30^. Thus, the overestimation of the SST response could be a result of an overestimation of the total freshwater forcing. Since the model calculates δ^18^O_sw_ prognostically, and obtains δ^18^O_c_ using a SST-based transfer function, the misrepresentation of δ^18^O_c_ is likely due to the SST overestimation. In turn, FWca yields the lowest RMSEs and best slopes across most tracers, with the exception of δ^18^O_sw_. FWli and FWul have the lowest regression slopes (${\upalpha}_{all}^{ul}<0.3$, ${\upalpha}_{all}^{li}<0.5$) and highest RMSEs of the four simulations.

The time series of the tracers confirm that FWpe overestimates both the long-term decrease in δ^18^O prior to 8 ka (Fig. 1b,f–i) and the cold SST anomalies (Fig. 1c–e). Analysis of the δ^18^O time series for the remaining simulations show the effect of each discharge in the early-Holocene proxy signal. The routing event in FWca reproduces most of the early-Holocene anomalies recorded between 8.5 and 8.3 ka, especially in the Labrador Sea SST and δ^18^O_sw_ (Fig. 1d,j), Gardar drift δ^18^O_c_ (Fig. 1h) and RAPiD subsurface (Fig. 1f). The magnitude of the short negative excursion in δ^18^O_ice_ at 8.2 ka in the GISP2 record is also best reproduced by FWca when compared to the other simulations (Fig. 1b). FWli also reproduces a sharp decrease in δ^18^O_ice_ at 8.2 ka (Fig. 1b), and in SST and δ^18^O_sw_ at Gardar drift (Fig. 1d,h), although it underestimates the magnitude of δ^18^O_ice_ anomalies at 8.2 ka. Finally, the remaining melting of LIS after its collapse simulated in FWul reproduces the stable low δ^18^O_c_ values at subsurface in the RAPiD core (Fig. 1f).

Thus, simulated δ^18^O shows that FWpe, FWca, FWli, and FWul reproduce different parts of the early Holocene signal. This suggests that a realistic freshwater flow for prompting the 8.2 ka event anomalies requires a combination of a long-term meltwater flux with a short-term flux intensification, possibly due to a change in routing of continental runoff and draining of Lake Agassis^21^.

## Hybrid simulations

**Figure 2:**
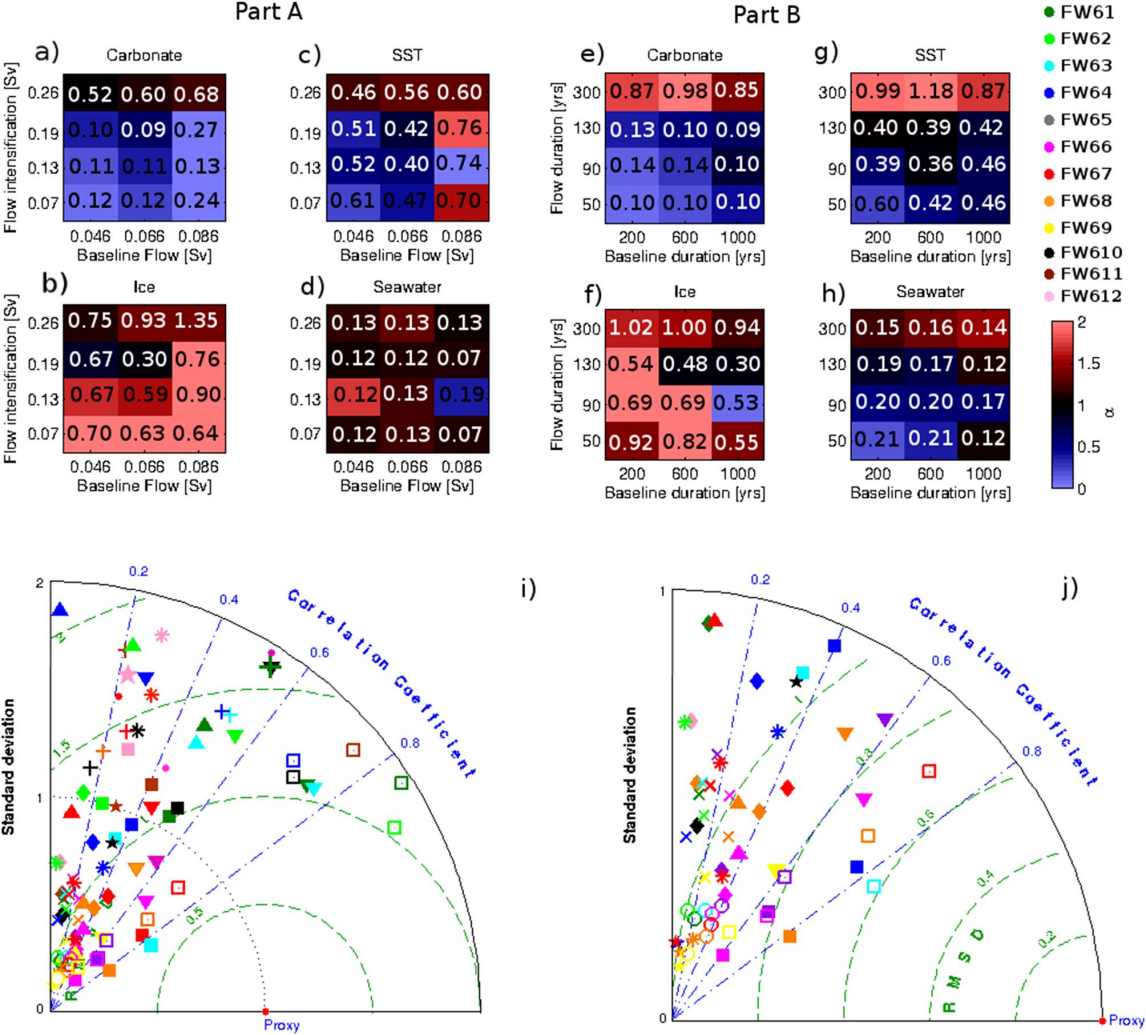
Analysis of simulations in Table 1—Parts A and B. (**a**) to (**h**) are the slope and RMSE values for each experiment. Plots (**a**–**d**) are for experiments in part A, while plots (**e**–**h**) are for experiments in part B. The colour of the squares represents the slopes according to the color bar, and RMSE values are indicated in the center of each cell. (**i**) Taylor diagram for comparison between proxy and simulated time series of δ^18^O anomalies: GISP ice core (filled square), Rapid Core δ^18^O_c_ in *G. inflata* (filled rhombus) and *G*. *bulloides* (plus)*,* Gardar Drift core δ^18^O_c_ for *G*. *bulloides* (filled circle), Florida Strait core δ^18^O_c_ for *G. rube*r (X), Gulf core δ^18^O_c_ for *G. ruber* (star), Rapid Core δ^18^O_sw_ in *G. inflata* (asterisk) and *G*. *bulloides* (filled triangle)*,* Gardar Drift core δ^18^O_sw_ (filled inverted triangle), Florida Strait core δ^18^O_sw_ (open circle), and δ^18^O_sw_ in Labrador Sea core (open square). The colors represent different simulations. Taylor diagram (**j**) is the same as (**i**), but zoomed in closer to the 0. Standard deviations are normalized by the core value, while RMSE is centered.

The simulations analyzed in this section (called hybrid hereafter) follow more complex freshwater release scenarios, testing the range of uncertainties in freshwater flux magnitude and duration as well as changes in freshwater forcing over time. In one set of the hybrid simulations (Table 1, Part A), the freshwater forcing is separated into two components: one lasts longer (1000 years background flux) with relatively low magnitudes (0.086 Sv, 0.066 Sv, and 0.046 Sv), while the other is shorter (130 year-long flux intensification) with relatively high magnitudes (0.13 Sv, 0.07 Sv, 0.19 Sv and 0.26 Sv). A comparison between the short fluxes in the Part A simulations (Fig. 2a–d) shows that a flux intensification of 0.19 Sv achieves the lowest RMSEs in δ^18^O_sw_, δ^18^O_c_ and δ^18^O_ice_ when compared with simulations with the same background flux but different short fluxes (Table S2). In turn, when comparing the long fluxes in the Part A simulations (Fig. 2a–d, columns), it is noticeable that the simulations with 0.066 Sv of background flux have the lowest RMSEs and slopes closest to 1 for SST, δ^18^O_c_ and δ^18^O_ice_. Thus, the comparisons based on RMSEs and slopes suggest that a background flux of 0.066 Sv and a short flux of 0.19 Sv best represent the tracer anomalies (simulation FW06—Table S2).

**Table 1:** Details of simulations used in this study.

(I) Reconstructions				
Experiment	Volume^a^	Duration	Flow (Sv)	References
FWpe	27.1	9–8 ka	0.086	Peltier^30^
FWca	8.2	8.5–8.2 ka	0.13	Carlson et al.^15^
FWli	5.3	8.31–8.18 ka	0.13	Li et al.^22^
FWul	9.5	8.2–7.6 ka	0.05	Ullmann et al.^18^

Duration	(II) Flux magnitude (Part A)			
9–8 ka	Sv	0.046	0.066	0.086
8.31–8.18 ka	0.26	FW10	FW11	FW12
	0.19	FW09	FW06	FW03
	0.13	FW07	FW04	FW01
	0.07	FW08	FW05	FW02

Flux	(III) Flux duration (Part B)			
0.066 Sv	Duration	200 years	600 years	1000 years
0.19 Sv	300 years	FW610	FW611	FW612
	130 years	FW67	FW64	FW61
	90 years	FW68	FW65	FW62
	50 years	FW69	FW66	FW63

The reconstructions table (I) describes the meltwater volumes, fluxes and durations for the homogeneous forcing experiments described in “Simulations based on earlier reconstructions” section. Experiments with hybrid freshwater forcing are separated into Part A and B (II and III). The long meltwater flux in the hybrid experiments in Part A have a fixed duration of 1000 years (9–8 ka), and the short flux is fixed at 130 years (8.31–8.18 ka). FW06 is the simulation in best agreement with proxy data in Part A, so the flux magnitudes of FW06 were used in Part B to test flux duration of the short flux. Note that FW06 is the same simulation as FW61. Volume (^a^) is in 10^5^ km^3^.

The Part B experiments were aimed at evaluating model sensitivity to the duration of the freshwater forcing. This was accomplished by adopting freshwater flux magnitudes from FW06, the experiment that best represented 8.2 ka event anomalies in Part A, and varying the durations of the individual phases of freshwater addition. The length of the shorter flux in this set of simulations varies from 50 to 300 years, while the longer flux varies from 200 to 1000 years (Table 1).

**Figure 3:**
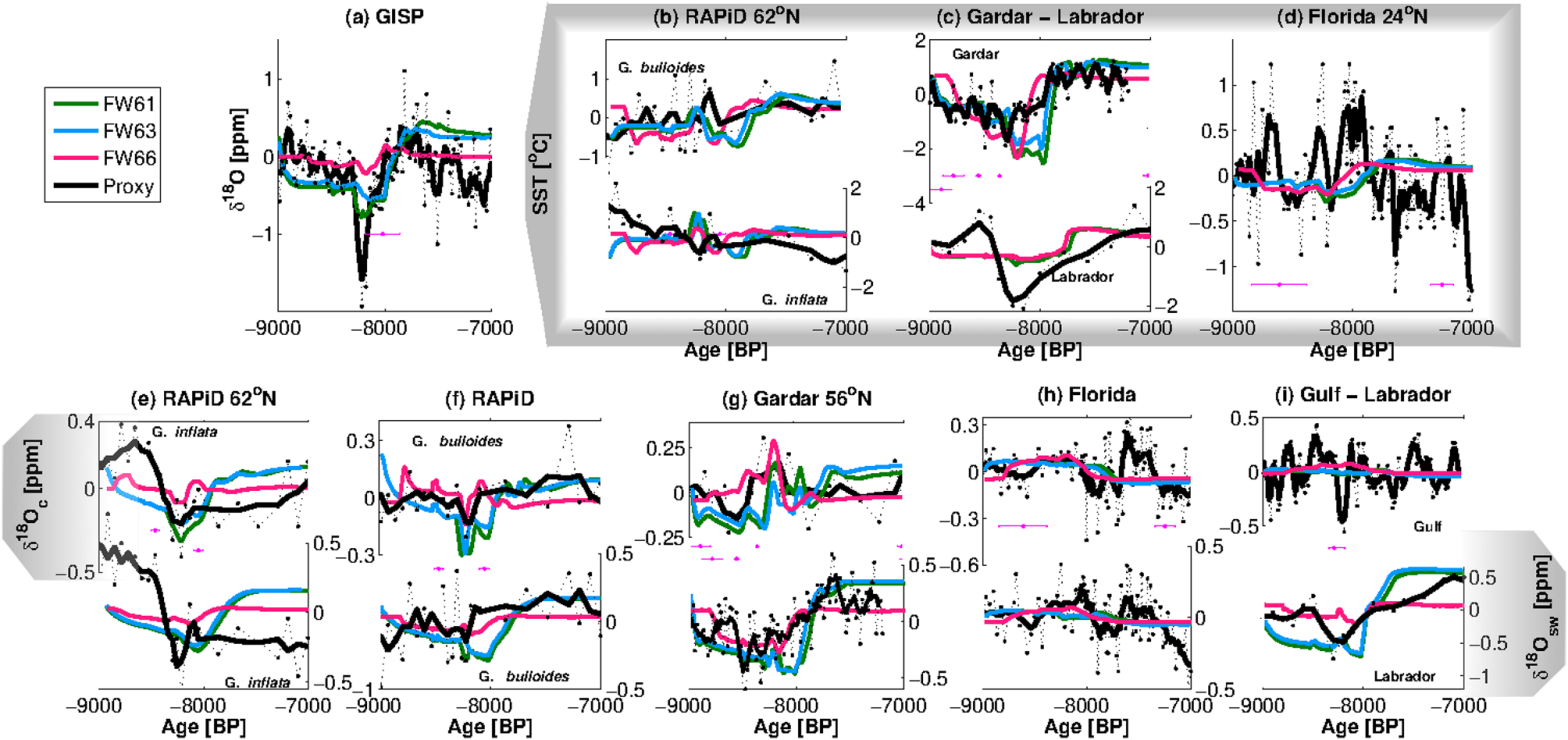
Comparison of simulated and reconstructed δ^18^O_sw_, δ^18^O_c_, δ^18^O_ice_ and SST time series for the three best fitting hybrid models: RAPiD (**b**,**e**,**f**), Gardar Drift (**c**,**g**), Florida Strait (**d**,**h**), GISP δ^18^O_ice_ (**a**), Gulf Strait δ^18^O_c_ (**i**-top) and Labrador Sea δ^18^O_sw_ (**i**-bottom) and SST (**c**-bottom). Black dashed and full lines are core values and 2-points moving average. Green, cyan, and magenta lines show FW61, FW63, and FW66 simulations, respectively. The pink horizontal crosses are the dating ( ) and dating errors ( ) for the proxies.

Simulations FW61 and FW63 show the best match with proxy data with slopes closest to 1 and consistently low RMSEs (Fig. 2e–h, Table S3). Further testing the similarity between the simulated and core time series of δ^18^O in a Taylor diagram allows for a more detailed comparison between simulations. All correlation values in the Taylor diagram are statistically significant (p < 0.05, n > 1000—Fig. 2i,j). The highest correlations for δ^18^O_sw_ are for the Labrador Sea cores and simulations FW61 (0.84), FW62 (0.88), and FW63 (0.83). In the Gardar Drift time series of δ^18^O_sw_, FW61 and FW63 have the strongest correlation with the core (both 0.75), but the lowest RMSE value is achieved by FW66 (0.76‰). FW61 and FW63 also have the highest positive correlations for δ^18^O_sw_ at the RAPiD core based on *G. bulloides* (0.47 and 0.48 respectively), for *G. bulloides* δ^18^O_c_ in the RAPiD core (0.54 for FW61). According to the Diebold-Mariano test, RMSEs for FW61, FW63 and FW66 in δ^18^O_c_ and δ^18^O_sw_ are significantly different with confidence varying from 85 to 99%. The exceptions are δ^18^O_c_ errors between FW61 and FW63, which are equal with 90% confidence. These results suggest that errors in δ^18^O_sw_ for simulations FW61, FW63 and FW66 are statistically different and possibly not random. Simulations FW61, FW63 and FW66 are the ones that best reproduce δ^18^O mean anomalies in most proxies and locations and have the best correlations and RMSEs for the whole time series. As a last step, and in an effort to determine the most realistic freshwater forcing, we will compare the time series produced by the three best fitting simulations with the proxy reconstructions at the six locations with high resolution data (Fig. 3).

Simulated δ^18^O_sw_ and δ^18^O_c_ for FW61/62/63 at the location of the RAPiD core now capture the magnitude of proxy anomalies for *G*. *bulloides* (Fig. 3f). FW61 and FW63 also reproduce the magnitude of the anomalies at the Gardar Drift core in the *G*. *bulloides* time series of δ^18^O_c_ and δ^18^O_sw_ (Fig. 3g), and the SST time series at the RAPiD location (Fig. 3b-top), and Gardar Drift (Fig. 3c-top).

GISP2 δ^18^O_ice_ is best simulated by FW61, which reproduces both the long-term oxygen isotope decrease and the timing of the short-lived decrease at 8.2 ka (Fig. 3a-top). Neither simulated δ^18^O_c_ in the Gulf of Mexico or SSTs in the Strait of Florida show significant variability (Fig. 3d,h,i-top). Taking into account that FW61 exhibits the best match to SST and δ^18^O in the proxy record, while also reproducing the GISP2 δ^18^O_ice_, we conclude that this simulation is the best representation of the 8.2 ka event in our study.

## Discussion

Our first set of simulations evaluate how well different freshwater sources to the North Atlantic reproduce ocean anomalies associated with the 8.2 ka event. Although FWca represents only one of these sources, i.e., a runoff routing event^15^, it yields the lowest RMSEs, best slopes and the best representation of most cores time series. This points to the routing event being one of the main contributors to the changes captured by the proxies during the early Holocene. Given that the regression slopes for FWul are significantly lower than for FWca, melting of the remaining LIS after its collapse (FWul) likely only played a background role in creating the climate anomalies at the 8.2 ka, while the routing event (FWca) had much more impact.

We then conducted several additional meltwater flux experiments in order to answer the following question: What magnitudes and rates of freshwater fluxes are most consistent with the 8.2 ka event proxy anomalies? The short 8.2 ka event anomalies recorded in δ^18^O climate archives are best reproduced with a simulation forced by a freshwater flux intensification of 0.19 Sv lasting for 130 years. This is in line with earlier simulations performed with the Community Climate System Model version 3, which reproduced the 8.2 ka SST anomalies with 0.13 Sv of freshwater discharge for 99 years^24^. Here, we show that a higher discharge estimate of 0.19 Sv embedded in a background flux of 0.066 Sv is able to reproduce δ^18^O anomalies in addition to SST anomalies.

**Figure 4:**
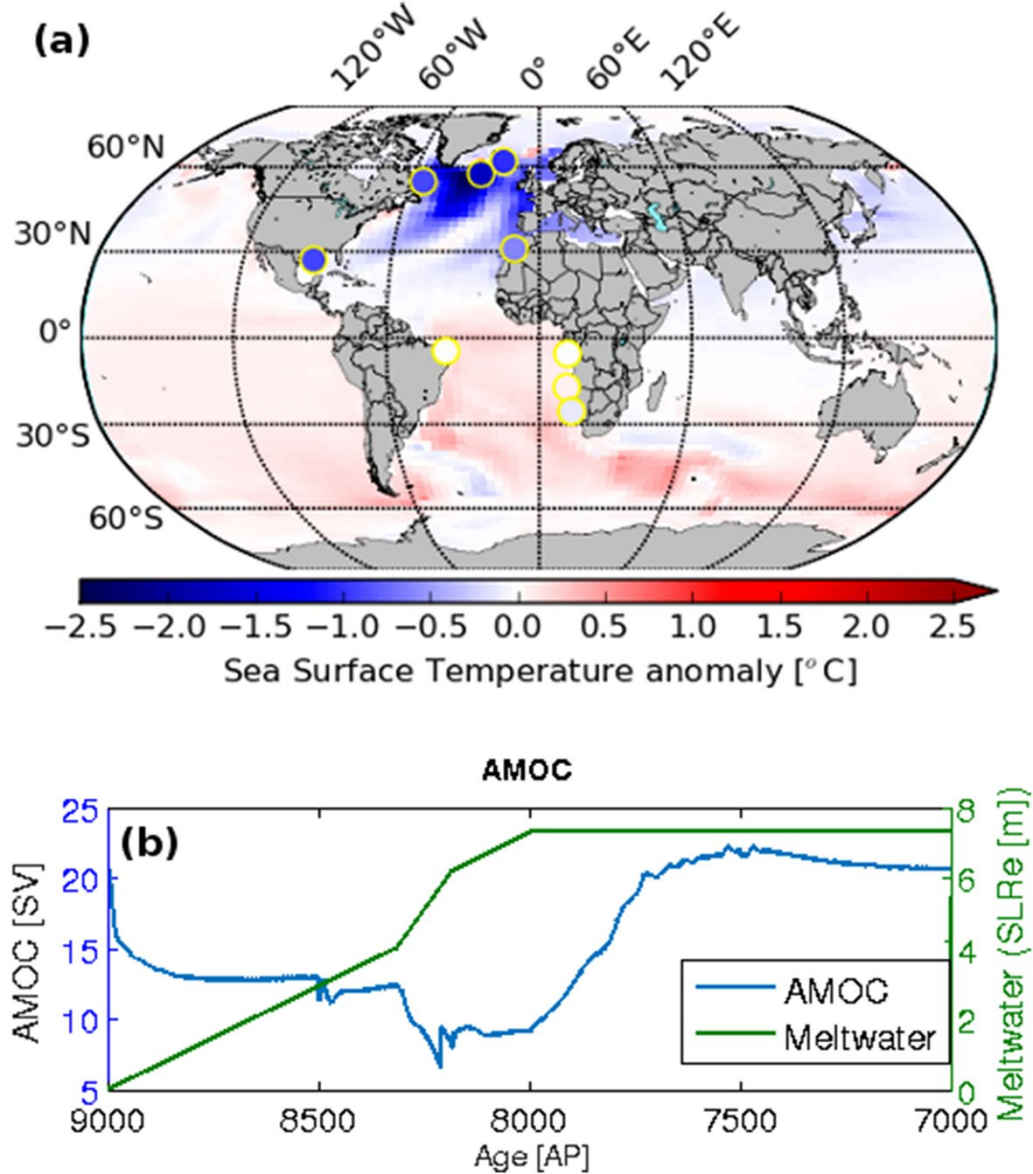
Climate impacts for the hybrid simulation FW61. (**a**) Proxy and model SST anomalies for the FW61 simulation. The color of the circles is plotted according to the anomaly value of the reconstructed SSTs. (**b**) Simulated maximum overturning streamfunction for the North Atlantic as a measurement for the Atlantic Meridional Overturning Circulation (right, blue line), and meltwater added in the FW61 experiment, in Sea Level Rise equivalent (SLRe, green line). Map (**a**) drawn by Wilton Aguiar on Python v2.7 (https://www.python.org/download/releases/2.7/).

Based on 35 δ^18^O and SST records from 27 different locations, we consider that our FW61 simulation was able to accurately reproduce the major trends and anomalies recorded in the proxy records for the 8.2 ka event and early-Holocene (Fig. 3). The FW61 simulation suggests that anomalies similar to those associated with the event could have been caused by a total meltwater addition of 7.5 m in SLR equivalent between 9–8 ka, with a short period of intensified flooding, equivalent to a SLR of 2.2 m (included in the 7.5 m estimate), between 8.31 and 8.18 ka (Figs. 4a,b, S2). This short intensification of the freshwater flux in FW61 has similar magnitude as the relative SLR in Southwest Scotland (1.45 m within 300 and 500 years)^31^, but the absolute value for our estimate is 0.75 m higher. This discrepancy could be explained by either local land uplift due to glacial isostatic adjustment over Scotland^32^, or by a combination of LIS melting and Canadian basin routing, since the routing would not contribute to eustatic SLR. The intensification in freshwater input of 2.2 m also matches previous eustatic SLR estimates from the Netherlands (3 ± 1.2 m within 200 years)^33^ and Mississippi delta (0.8–2.2 m within 130 years)^22^. Estimates of SLR rates on longer time-scales for the early-Holocene however differ considerably from ours. Rates of 17.9 mm year^−1^ (8600–7100 BP), and 24 mm year^−1^ (extending up to 8948–8206 BP) are recorded on the coast of Germany^34^ and Norway^35^, much higher than our 7.5 mm year^−1^ estimate. Because the melting of Antarctic Ice Sheet contributed to less than 3 cm of SLR in early-Holocene^51^, this difference in meltwater fluxes likely derives from LIS additional melting. This is expected since the meltwater volume in this study is an estimate of the meltwater that was added to the Labrador Sea, part of which was then advected to deep water formation sites, thus affecting large-scale ocean circulation and climate. Additionally, meltwater from the LIS in the early-Holocene was discharged into wide regions in the Arctic and North Atlantic and thus account for a total volume higher than the one we find here^30^. Therefore, our estimate does not represent the total LIS melting and corresponding SLR for this time span. Neither FW61 nor the proxy records show a clear 8.2 ka response in the Florida Strait (SST). The 8.2 ka event might therefore not have had a significant and far-reaching impact on the Florida Strait region, causing a climate response within model or proxy data background variability. A simulation of the 8.2 ka event with the Hadley Centre Coupled Model, version 3 (HadCM3) also did not reproduce any measurable SST anomalies in the Gulf of Mexico^36^, thus suggesting that the core SST signal in that location is likely not due to meltwater forcings involved in the 8.2 ka event. The AMOC response to the 8.2 ka freshwater forcing is still debated in the scientific community. In our best-fitting FW61 simulation, AMOC weakens by 62% (13 Sv, Fig. 4b) without collapsing, supporting earlier evidence of substantial AMOC weakening without a collapse during the 8.2 ka event^37,38^. Matero et al.^36^ find that AMOC weakens by 55% of its initial overturning, similar to our estimate, based on simulations with the HadCM3.

## Implications

The magnitude of the simulated climate change during the 8.2 ka event offers a pertinent reference point for future climate trends^12^. The Greenland Ice Sheet is undergoing considerable melting and this is likely to continue well into the future^1,29^. Greenland melting scenarios for the next millennium project SLR of 7.28 m for the RCP8.5 scenario of the Intergovernmental Panel on Climate Change^39^. Current meltwater fluxes from the Greenland Ice Sheet are estimated to be ~ 0.005 Sv^29^. Even though this flux is considerably smaller than the ones used in our experiments, projections of freshwater flux intensification for the next centuries are similar to the FW61 baseline flows. For example, Golledge et al.^6^ found an increase in freshwater flux from Greenland ice-sheet melting of 0.015 Sv by 2100 in the RCP8.5 scenario. Lenaertes et al.^40^ project an acceleration of the meltwater flux from Greenland up to 0.08 ± 0.003 Sv by 2200, while the maximum melting scenario of Aschwanden et al.^39^ projects a flux exceeding 0.17 Sv by 2300 (15 mm of SLR year^−1^). Bakker et al.^4^ found a median discharge higher than 0.08 Sv by the year 2300. The projected input of freshwater into the North Atlantic associated with the RCP8.5 scenario is therefore of the same magnitude as those in the FW61 simulation in terms of total SLR contribution (7.5 m), duration (1000 years) and flux magnitude (0.066 SV to 0.19 SV). However, it is important to highlight that future emission scenarios also include intensive surface radiative warming, which will add to the stratification effect and thus intensify the future overturning weakening^28^. Additionally, future GIS meltwater will likely flow into the coastal areas surrounding the ice sheet, instead of exclusively into the Labrador Sea^39^, and thus, its impact on the ocean overturning will potentially differ from the focused meltwater injection in the Labrador during the 8.2 ka event. Moreover, the climate response to an increase in meltwater will be in addition to the much greater warming response due to increasing greenhouse gas concentrations, as well as changes due to topography and albedo changes over Greenland. Nevertheless, the estimated meltwater flux from the GIS in the not too distant future is comparable to the fluxes we find as the forcing behind the 8.2 ka event.

## Methods

### Model and data

Simulations were performed using the University of Victoria Earth System Climate Model version 2.9 (UVic Model)^41^, with the addition of oxygen isotopes^42,43^. Water in the ocean, atmosphere, sea-ice, and on land is compartmentalized into ^18^O and ^16^O to allow the estimation of δ^18^O distribution^42–46^. A detailed description of the experimental setup is given in the Supporting Information. We compared simulated δ^18^O and SSTs to paleoclimate record mean anomalies for the 8.2 ka event (averaged between 7.9 and 8.5 ka—Supplementary) and time series from six locations (Fig. S1-stars). Mean anomalies of oxygen isotope ratios in seawater (δ^18^O_sw_), carbonate (δ^18^O_c_), ice (δ^18^O_ice_), and SSTs, were taken from Morrill et al.^47^. These proxy anomalies are based on data from 27 cores (Fig. S1), some recording more than one paleoclimate proxy. Overall, our analysis includes ten SST records, ten δ^18^O_sw_ records, seven δ^18^O_c_ records and eight δ^18^O_ice_ records. The mean anomalies in the simulations are calculated following the methodology by Morrill et al.^47^. They are defined as the difference between SST (or δ^18^O) values averaged between 7.9 and 8.5 ka and their climatological mean, only for values above (or below) the mean plus (minus) two standard deviations. The climatological mean is defined as the average between 9 and 7 ka, excluding the period between 7.9 and 8.5 ka.

For most records, the simulated values were taken at the model’s grid cell closest to the geographical coordinates of each core, at the surface level of the ocean model (17.5 m). The tracers reconstructed from *Globorotalia inflata* were compared to the simulated ocean tracers averaged between 82.5 and 177.5 m, due to the wide range of vertical migration inherent to this species. Thus, time series for the RAPiD core based on *Globigerina bulloides* reflect surface changes, while those based on *G. inflata* reflect changes in the upper thermocline. The UVic model does not simulate isotopic fractionation during foraminiferal calcification. Thus, model δ^18^O_c_ was estimated by an SST-based transfer function^48,49^.

In order to evaluate the simulations’ skill in reproducing the reconstructed δ^18^O, the linear regression’s slope (⍺) and Root Mean Square Errors (RMSE) were calculated for the model anomalies using proxy anomalies as reference. Equality between model and proxy happens when ⍺ = 1. For the time series, centered RMSE, normalized standard deviations and Pearson’s correlations were compared in a Taylor diagram in order to evaluate the performance of the simulations in reproducing the proxy time series. To assure that the difference of the RMSEs for the time series of δ^18^O_sw_ and δ^18^O_c_ are significant, we performed a Diebold-Mariano test^50,^^51^ between each of the experiments in Part B. We then report the Diebold-Mariano test results and its significance level for the simulations with the best performances. All remaining values of the Diebold-Mariano test and its critical confidence percentages are presented in the Supplementary Information (Supplementary S2). More information on the experimental setup and core data can be found in the Supplementary Material.

### Freshwater forcing for the simulations based on earlier reconstructions

There are four main estimates of freshwater input into the North Atlantic close to the time of the 8.2 ka event. A glacial isostatic adjustment model by Peltier^30^ estimates that 27.1 × 10^5^ km^3^ of freshwater were added to the North Atlantic from LIS retreat from 9 to 8 ka. The meltwater from LIS estimated by Peltier^30^ did not flow entirely into the Labrador Sea, so this estimate can be used as an upper constraint for total meltwater discharged in the Labrador Sea in the period. Carlson et al.^15^ estimate a 0.13 Sv ± 0.03 Sv increase in the inflow of freshwater into Labrador Sea after the collapse of Hudson Bay that ended ~ 8.2 ka^18^ due to the routing of the western Canadian Plains runoff (8.2 × 10^5^ km^3^ in volume). Although the routing event does not contribute to SLR, it would still alter the oxygen isotope ratios and surface water buoyancy in the Labrador Sea, thus potentially affecting deepwater formation rates. Li et al.^22^ found a 1.5 ± 0.7 m of eustatic SLR between 8.31 ka and 8.18 ka (5.3 × 10^5^ km^3^ in volume) from a SLR reconstruction, which includes the freshwater release from the lake outburst. Ullman et al.^18^ estimate that additional melting of the LIS after its collapse contributed to 3.6 ± 0.4 m of SLR that began ~ 8.2 ka and ended 7.6 ± 0.6 ka (~ 9.5 × 10^5^ km^3^ in volume). The estimated Antarctic Ice Sheet contribution to SLR during the early-Holocene is lower than 3 cm, i.e. substantially smaller than LIS^51^, so no meltwater was added in the Southern Hemisphere in the simulations. Using these estimates, we derived four main freshwater release experiments running from 9 ka until 7 ka (Table 1, (I) Reconstructions).

It is important to highlight that the four freshwater release estimates refer to different processes, and thus each simulation will represent the effect of a specific process in creating proxy anomalies of the 8.2 ka event: FWca represents the Canadian Plains routing event, FWul represents the effect of meltwater from the remaining LIS after its collapse, FWli represents the effect of the total freshwater addition to the ocean surrounding the 8.2 ka event (not accounting for routing events), and FWpe represents the total early-Holocene meltwater from the LIS. By simulating these separately, we estimate the signature of each process on the δ^18^O and SST records.

In our simulations, all freshwater was added to the Labrador Sea (50°N–65°N; 70°W–35°W). Meltwater from the LIS and Lake Agassiz are estimated to have had a δ^18^O varying from − 24 to − 25‰ during the early-Holocene^52,53^; we therefore added freshwater with a δ^18^O of − 25‰. Overturning in FWpe collapsed after 8 ka; to restart the North Atlantic deep convection smoothly a virtual salt flux decreasing from − 0.2 to − 0.05 Sv (8 ka until 7.5 ka) with no isotopic signature was added.

### Freshwater forcing of the hybrid scenarios

In addition to this first set of simulations, which are based on earlier geological reconstructions and described in “Freshwater forcing for the simulations based on earlier reconstructions” section, we also integrated additional sensitivity simulations. Twenty-four experiments were performed based on the uncertainty ranges of the Peltier^30^, Li et al.^22^, and Carlson et al.^15^ estimates (Table 1). The 7.5 m in SLR equivalent estimated by Peltier^30^ was not fully released into the Labrador Sea. In turn, Li et al.^30^ estimated the date of the meltwater outburst within 8.245 ± 0.065 ka and their flux estimate has a 0.06 Sv uncertainty. Additionally, the Canadian continental basin routing event from Carlson et al.^15^ likely contributed to an enhancement of freshwater flow to the Labrador Sea of 0.13 Sv lasting up to 300 years. Together, these result in potential freshwater fluxes varying between 0.046 and 0.26 Sv and lasting between 200 and 1000 years. With these experiments, called “hybrid”, we test a more complex meltwater flux scenario, based on a background freshwater forcing over a longer time period, a rerouting event and a shorter pulse, more intensive, drainage event. Both the magnitude of the meltwater fluxes (Part A), and their duration (Part B) are tested. Finally, a 2.5 SV freshwater flow to the Labrador Sea was added at year 8.47 ka in all simulations in order to simulate the Lake Agassiz outburst^19^. The exact date of the Lake Agassiz collapse is uncertain due to uncertainties on reservoir ages of marine cores, which precludes further exploration of the date of the collapse in the simulations in this study.

## Supplementary Information

Supplementary Information 1.

Supplementary Information 2.

## Data Availability

Simulated data for this research is available in the Zenodo database (https://doi.org/10.5281/zenodo.4282563) and by contact to the first author. The core data used is available in these in-text data citation references: Morrill et al.^10^, Peltier^29^, Carlson et al.^14^, Li et al.^21^, Ullmann et al.^17^. Remaining data not present in these sources are available in the supplementary material.
