# Supplementary material for: Magnitude of the 8.2 ka event freshwater forcing based on stable isotope modelling and comparison to future Greenland melting
Source: Sci Rep. 2021 Mar 9;11:5473. doi: 10.1038/s41598-021-84709-5 (PMC7943769; doi:10.1038/s41598-021-84709-5)
Supplement: Supplementary file 1 — Supplementary Information 1. [file 41598_2021_84709_MOESM1_ESM.docx]

Supporting Information for

**Magnitude of the 8.2 ka event freshwater forcing based on stable isotope modelling and comparison to future Greenland melting**

Wilton Aguiar^1,*^, Katrin J. Meissner^2^, Alvaro Montenegro^3^, Luciana F. Prado^4,5^, Ilana Wainer^4^, Anders E. Carlson^6^, and Mauricio M. Mata^1^

^1^Laboratório de Estudos dos Oceanos e Clima, Instituto de Oceanografia, Universidade Federal do Rio Grande – FURG. Rio Grande, RS, 96203-900, Brazil.

^2^Climate Change Research Center and ARC Centre of Excellence for Climate Extremes, University of New South Wales, Sydney, Australia.

^3^Atmospheric Sciences Program, Department of Geography, The Ohio State University, Columbus, Ohio.

^4^Instituto Oceanografico, Universidade de Sao Paulo, Sao Paulo, 05508-120, Brazil.

^5^Instituto de Geociências, Universidade de Brasília, Brasília, 70297-400, Brazil.

^6^Oregon Glaciers Institute, Corvallis, OR, USA

*aguiar.wilton@gmail.com

**Contents of this file**

Figure S1, Figure S2, Table S1, Table S2, Table S3

**Introduction**

This supplementary material shows the location and details of cores used, gives specific information about the simulations, and the climate signals found for the 8.2 ka.

**Reconstructed tracers**

A total of 35 paleorecords were used to compare the Sea Surface Temperature (SST) and δ^18^O with simulations, with five of the cores having high enough resolution to provide time series (Fig S1).

**
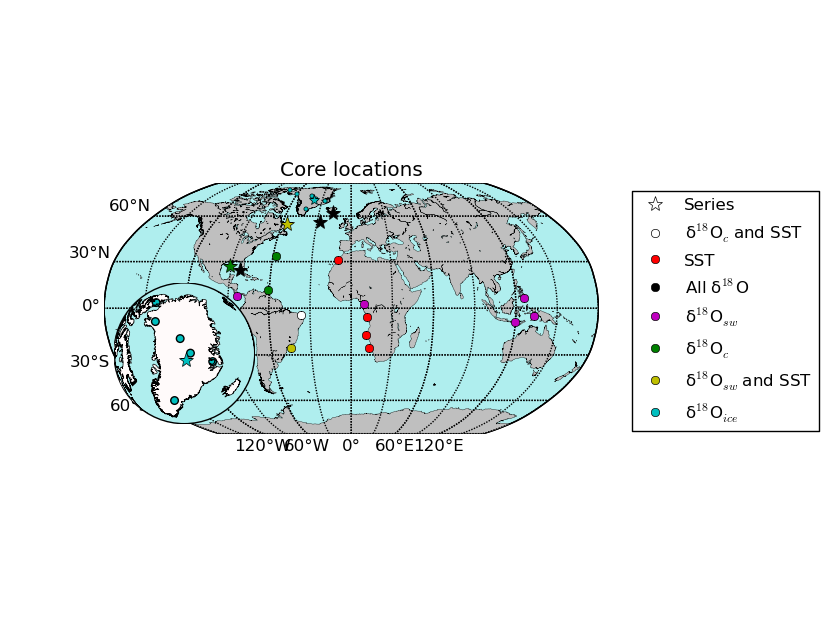
**

**Figure S1.** Location of cores used in this study. Circles are cores used in mean anomalies, while stars are cores used for time series analysis. The color legend is for both circles and stars. The cores in black also record SSTs. Drawn by Wilton Aguiar using Python 2.7 (https://www.python.org/download/releases/2.7/)

The mean proxy anomalies in SST, δ^18^O_sw_, δ^18^O_c_, δ^18^O_ice_ were obtained from Morrill et al., (2013)^1^, while the time series used are described in table S1. Mean simulated anomalies are calculated as the mean value of the tracer between 7.9 ka and 8.5 ka for values surpassing 2 standard deviations from the climatological mean for the early-Holocene. The climatological mean here is defined as the mean SST or δ^18^O from 9 to 7 ka, with a hiatus between 8.5 ka and 7.9 ka to avoid redundancy. This mean anomaly calculation method is the same applied by Morrill et al. (2013)^1^. There are three species of foraminifera in the cores used to derive the time series: *Globorotalia inflata*, *Globigerina bulloides*, and *Globigerinoides ruber*. Both *Globigerina bulloide*s and *Globigerinoides ruber* occupy the mixed layer (upper 50 m) being appropriate sources of information for SST reconstructions. *Globorotalia inflata* migrates vertically up to 200 m, and calcifies within the seasonal thermocline, thus better representing sub-surface temperatures^2^. SST and δ^18^O derived from *G. bulloides* and *G. rube*r were compared with the model SST and δ^18^O in the uppermost layer (17 m). *G. inflata* derived SST and δ^18^O were compared with the simulated tracers averaged between 82.5 m and 177.5m. δ^18^O_ice_ in the Greenland Ice Sheet Project ice core^3^ (GISP) was compared to the oxygen isotope ratio in the simulated surface ice at the same location in Greenland. Simulated tracer values from both the mean anomalies and timeseries are taken from the model latitudinal and longitudinal grid points closest to each core location.

**University of Victoria Earth System Climate Model (UVic model)**

Simulations were performed using the University of Victoria Earth System Climate Model version 2.9, with the addition of oxygen isotopes^11,12^. The UVic model is coupled with seven main components: vegetation, sea ice, sediment, atmosphere, land surface, and ocean components. The ocean component is the Modular Ocean Model Version 2, which has 3.6^o^x1.8^o^ of longitudinal and latitudinal resolution, and comprises of 19 vertical levels with spacing ranging from 500 m in the deepest levels to 50 m at the surface^4^.

**Table S1.** Time series locations and source cores.

| **Core** | **Latitude, Longitude** | **Source** | **Variables** | **Reference** |
| --- | --- | --- | --- | --- |
| GISP | 72.6°N, 38.5°W | Ice | δ^18^O_ice_ | Grootes et al., (1997)^3^ |
| RAPiD core | 62.1°N, 17.8°W | G. *inflata* | δ^18^O_c_ and δ^18^O_sw_ | Thornalley et al (2009)^5^ |
|  |  | G. *bulloides* | δ^18^O_c_ and δ^18^O_sw_ |  |
| Gardar Drift | 57.4°N,27.9°W | G. *bulloides* | δ^18^O_c_ and δ^18^O_sw_ | Elmore et al., (2014)^6^ |
| Florida Strait | 24.3°N,83.3°W | G. *ruber* | δ^18^O_c_ and δ^18^O_sw_ | Schmidt et al., (2011)^7^ |
| Gulf of Mexico | 26.9°N,91.3°W | *G. ruber* | δ^18^O_c_ | Nuernberg et al., (2008)^8^ |
| Labrador Sea | 54.6°N,56.2°W | N. *pachyderma* | SST and δ^18^O_sw_ | Hoffman et al., (2012)^9^ |

A ten thousand years long equilibrium simulation was performed using 9 ka orbital parameters, i.e. eccentricity=0.0167, obliquity=23.45^o^, and [CO_2_]=280 ppmv, and initial pre-industrial δ^18^O_sw_ concentration, in order to assure full equilibrium of ocean parameters and circulation. All freshwater forcing simulations depart from the equilibrium run. Finally, the simulated time series of δ^18^O_c_ were converted from δ^18^O_sw_ and SST using the equation from Bemis et al., (1998)^10^:

δ^18^O_c_ (ppmv) = δ^18^O_sw_ (ppmv) + [T(^o^C) - 13.2] /4.89

**Statistics from hybrid experiments**

Tables S2 and S3 present the values for the Root Mean Square Error (RMSE) and slopes (𝛼) for part A and B experiments.

**Table S2.** Centered Root mean square errors and slopes (𝛼) for the simulations in part A experiments. RMSEs closest to 0 and slopes closest to 1 are highlighted in red.

| **Part A experiments** | | | δ^18^O_sw_ | | δ^18^O_c_ | | δ^18^O_ice_ | | SST | |
| --- | --- | --- | --- | --- | --- | --- | --- | --- | --- | --- |
| Short flux (SV) | Long flux (SV) | Simulation | RMSE | 𝛼 | RMSE | 𝛼 | RMSE | 𝛼 | RMSE | 𝛼 |
| 0.13 | 0.086 | FW01 | 0.19 | 0.35 | 0.13 | 0.02 | 0.9 | 1.98 | 0.74 | 0.04 |
| 0.07 |  | FW02 | ***0.07*** | ***1.1*** | 0.24 | -0.26 | 0.64 | 4.98 | 0.70 | 1.57 |
| 0.19 |  | FW03 | ***0.07*** | ***1.1*** | 0.27 | -0.59 | 0.76 | 6.1 | 0.76 | 1.8 |
| 0.13 | 0.066 | FW04 | 0.13 | 1.2 | ***0.11*** | 0.38 | 0.59 | 1.55 | ***0.40*** | 0.58 |
| 0.07 |  | FW05 | 0.13 | 1.2 | 0.12 | 0.21 | 0.63 | 1.95 | ***0.47*** | 0.41 |
| 0.19 |  | FW06 | ***0.12*** | ***1.1*** | ***0.09*** | ***0.63*** | ***0.30*** | ***0.9*** | ***0.42*** | ***0.79*** |
| 0.13 | 0.046 | FW07 | ***0.12*** | 1.7 | ***0.11*** | ***1.1*** | 0.67 | 1.65 | 0.52 | 0.2 |
| 0.07 |  | FW08 | ***0.12*** | ***1.1*** | 0.12 | 0.16 | 0.70 | 2.0 | 0.61 | 0.52 |
| 0.19 |  | FW09 | ***0.12*** | ***1.08*** | ***0.10*** | 0.05 | 0.67 | ***0.8*** | 0.51 | 0.38 |
| 0.26 | 0.046 | FW10 | 0.13 | 1.15 | 0.52 | 0.36 | 0.75 | ***1.1*** | ***0.46*** | ***1.2*** |
| 0.26 | 0.066 | FW11 | 0.13 | 1.33 | 0.60 | ***1.2*** | 0.93 | 1.37 | 0.56 | 1.34 |
| 0.26 | 0.086 | FW12 | 0.13 | ***1.05*** | 0.68 | ***1.2*** | 1.35 | 1.37 | 0.60 | 1.32 |

**Table S3.** Centered Root mean square errors and slopes (𝛼) for the simulations in part B experiments. RMSEs closest to 0 and slopes closest to 1 are highlighted in red.

| **Part B Experiments** | | | δ^18^O_sw_ | | δ^18^O_c_ | | δ^18^O_ice_ | | SST | |
| --- | --- | --- | --- | --- | --- | --- | --- | --- | --- | --- |
| Short flux (yrs) | Long flux (yrs) | Simulation | RMSE | Slope | RMSE | Slope | RMSE | Slope | RMSE | Slope |
| 130 | 1000 | FW61 | ***0.12*** | ***1.1*** | ***0.09*** | ***0.63*** | ***0.30*** | ***0.9*** | ***0.42*** | ***0.79*** |
| 90 |  | FW62 | 0.17 | 0.55 | ***0.10*** | ***0.75*** | ***0.53*** | 0.05 | ***0.46*** | **0.67** |
| 50 |  | FW63 | ***0.12*** | ***1.06*** | ***0.10*** | 0.43 | ***0.55*** | 1.41 | ***0.46*** | **0.67** |
| 130 | 600 | FW64 | 0.17 | 0.73 | ***0.10*** | 0.51 | ***0.48*** | ***0.92*** | ***0.39*** | ***0.96*** |
| 90 |  | FW65 | 0.20 | 0.56 | 0.14 | 0.26 | 0.69 | 2.51 | ***0.36*** | ***0.90*** |
| 50 |  | FW66 | 0.21 | 0.37 | ***0.10*** | 0.17 | 0.82 | 2.46 | ***0.42*** | 0.52 |
| 130 | 200 | FW67 | 0.19 | 0.82 | 0.13 | 0.36 | ***0.54*** | 2.28 | ***0.40*** | 01.03 |
| 90 |  | FW68 | 0.20 | 0.52 | 0.14 | 0.10 | 0.69 | 2.25 | ***0.39*** | 0.58 |
| 50 |  | FW69 | 0.21 | 0.17 | ***0.10*** | 0.05 | 0.92 | 1.4 | 0.60 | 0.21 |
| 300 |  | FW610 | 0.15 | 1.22 | 0.87 | 1.78 | 1.02 | 1.6 | 0.99 | 1.94 |
|  | 600 | FW611 | 0.16 | 1.35 | 0.98 | 02.03 | 1.00 | 1.52 | 1.18 | 2.15 |
|  | 1000 | FW612 | 0.14 | 1.54 | 0.85 | 1.43 | 0.94 | 1.2 | 0.87 | 1.73 |

**Freshwater Budget in simulation FW61**

The anomalies in precipitation and river discharge for the simulation FW61 exhibit only negative values between 8.4 ka and 8.2 ka (Fig S2). The balance between precipitation, evaporation, and runoff over the North Atlantic (north of 30^o^N - Fig S2) yields a maximum freshwater anomaly of -0.015 Sv, and is thus six times lower than the low background flux (0.066 Sv) in FW61. Hence, no significant volume of freshwater is added or removed to/from the North Atlantic Ocean through the prognostic freshwater budget in the model (i.e. simulated precipitation + simulated runoff - simulated evaporation), when compared with the forced freshwater anomalies (See Methods in the main manuscript).

**Figure S2.** The North Atlantic freshwater budget in the North Atlantic and over North America. (a) The contour of anomalies in precipitation minus evaporation (P-E) and continental runoff discharge (R) in kg m^-2^ s^-1^, calculated as the difference of the mean variable between 8.4 ka - 8.2 ka and between 9 ka - 7 ka. The colormaps inside the yellow contour represent the anomalies in river discharge, while the colormaps outside the yellow contours are for anomalies in P-E. (b) Anomalies of the freshwater budget in the North Atlantic (north of 30^o^N: precipitation - evaporation + river runoff) in Sv, relative to the mean freshwater budget from 9ka - 7ka. Drawn by Wilton Aguiar using Matlab R2013a (https://www.mathworks.com/products/matlab.html)
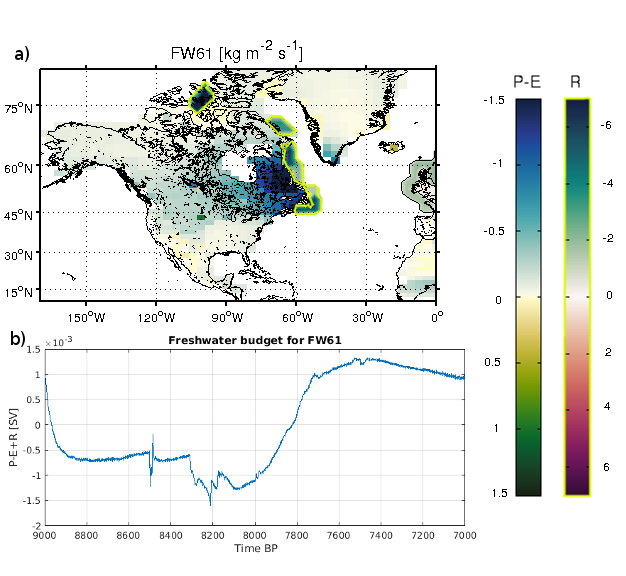


**References**

**1.**Morrill, C. et al. Proxy benchmarks for intercomparison of 8.2 ka simulations. *Clim. Past.* **9**, 1; 10.5194/cp-9-423-2013 (2013).

**2.**Ganssen, G. M. & Kroon, D. The isotopic signature of planktonic foraminifera from NE Atlantic surface sediments: implications for the reconstruction of past oceanic conditions. *J. Geol. Soc. Lond.* **157**, 693–699 (2000).

**3.**Grootes, P.M., and M. Stuiver. Oxygen 18/16 variability in Greenland snow and ice with 10^3 to 10^5-year time resolution. *Jour. Geoph. Re.* 102:26455-26470 (1997).

**4.**Pacanowski, R.C. *et al*. The GFDL modular ocean model user guide. *Geoph. Fluid Dyn. Lab. Tech. Rep.* **2**, 17. [Available from NOAA/Geophysical Fluid Dynamics Laboratory, P.O. Box 308, Princeton, NJ 08542-0308.] (1991).

**5.**Thornalley, D.J., Elderfield, H. and McCave, I.N. Holocene oscillations in temperature and salinity of the surface subpolar North Atlantic. *Nature*. **457**, 7230. 10.1038/nature07717 (2009).

**6.**Elmore, A.C., Wright, J.D. and Southon, J. Continued meltwater influence on North Atlantic Deep Water instabilities during the early Holocene. *Mar. Geol.* **360**. 10.1016/j.margeo.2014.11.015, (2015).

**7.**Schmidt, M.W. and Lynch‐Stieglitz, J. Florida Straits deglacial temperature and salinity change: Implications for tropical hydrologic cycle variability during the Younger Dryas. *Paleocean.* **26**, 4. 10.1029/2011PA002157 (2011).

**8.**Nuernberg, D., M. *et al*. Interacting Loop Current variability and Mississippi River discharge over the past 400 kyr. *Earth Planet. Sci. Lett.* **272**, 1-2. (2008).

**9.**Hoffman, J.S. *et al*. Linking the 8.2 ka event and its freshwater forcing in the Labrador Sea. *Geophys. Res. Lett*. **39**, L18703. 10.1029/2012GL053047. (2012).

**10.**Bemis, B.E. *et al*. Reevaluation of the oxygen isotopic composition of planktonic foraminifera: Experimental results and revised paleotemperature equations. *Paleoceanography*. **13**, 2; 10.1029/98PA00070 (1998).

**11.**Weaver, A.J. *et al*. The UVic Earth System Climate Model: Model description, climatology, and applications to past, present and future climates. *Atmosphere-Ocean*. **39**, 4; 10.1080/07055900.2001.9649686 (2001).

**12.**Brennan, C.E., Weaver, A.J., Eby, M. & Meissner, K.J. Modelling oxygen isotopes in the University of Victoria Earth System Climate Model for pre-industrial and Last Glacial Maximum conditions. *Atmosphere-ocean*. **50**, 4; 10.1080/07055900.2012.707611 (2012).
